# Spatial model of *Dengue* Hemorrhagic Fever (DHF) risk: scoping review

**DOI:** 10.1186/s12889-023-17185-3

**Published:** 2023-12-07

**Authors:** Ririn Pakaya, D. Daniel, Prima Widayani, Adi Utarini

**Affiliations:** 1 Doctoral Program in Public Health, Faculty of Medicine, Public Health and Nursing, Universitas Gadjah Mada, Yogyakarta, Indonesia; 2 Department of Public Health, Public Health Faculty, Universitas Gorontalo, Gorontalo, Indonesia; 3 Department of Health Behaviour, Environment and Social Medicine, Faculty of Medicine, Public Health and Nursing, Universitas Gadjah Mada, Yogyakarta, Indonesia; 4 Department of Geographic Information Science, Faculty of Geography, Universitas Gadjah Mada, Yogyakarta, Indonesia; 5 Department of Health Policy and Management, Faculty of Medicine, Public Health and Nursing, Universitas Gadjah Mada, Yogyakarta, Indonesia

**Keywords:** Dengue, Risk factor, Outbreaks, Scoping review

## Abstract

**Background:**

Creating a spatial model of dengue fever risk is challenging duet to many interrelated factors that could affect dengue. Therefore, it is crucial to understand how these critical factors interact and to create reliable predictive models that can be used to mitigate and control the spread of dengue.

**Methods:**

This scoping review aims to provide a comprehensive overview of the important predictors, and spatial modelling tools capable of producing Dengue Haemorrhagic Fever (DHF) risk maps. We conducted a methodical exploration utilizing diverse sources, i.e., PubMed, Scopus, Science Direct, and Google Scholar. The following data were extracted from articles published between January 2011 to August 2022: country, region, administrative level, type of scale, spatial model, dengue data use, and categories of predictors. Applying the eligibility criteria, 45 out of 1,349 articles were selected.

**Results:**

A variety of models and techniques were used to identify DHF risk areas with an arrangement of various multiple-criteria decision-making, statistical, and machine learning technique. We found that there was no pattern of predictor use associated with particular approaches. Instead, a wide range of predictors was used to create the DHF risk maps. These predictors may include climatology factors (e.g., temperature, rainfall, humidity), epidemiological factors (population, demographics, socio-economic, previous DHF cases), environmental factors (land-use, elevation), and relevant factors.

**Conclusions:**

DHF risk spatial models are useful tools for detecting high-risk locations and driving proactive public health initiatives. Relying on geographical and environmental elements, these models ignored the impact of human behaviour and social dynamics. To improve the prediction accuracy, there is a need for a more comprehensive approach to understand DHF transmission dynamics.

**Supplementary Information:**

The online version contains supplementary material available at 10.1186/s12889-023-17185-3.

## Introduction

Dengue Haemorrhagic Fever (DHF) is a viral disease spread by the female Aedes mosquito, specifically the Aedes aegypti. DHF has become a major public health concern in tropical and subtropical countries around the world in recent years [1]. It is estimated that approximately 390 million infections occur each year, with approximately 3.9 billion people at direct risk of the disease [2, 3]. Aedes aegypti bites humans to spread DHF, commonly known as break-bone fever, after ingesting blood. The disease is swiftly spread over the world by it [4, 5]. The number of DHF cases reported to WHO increased 8 folds over the last two decades, from 505,430 cases in 2000 to over 2.4 million in 2010, and 5.2 million in 2019. Reported deaths between the years 2000 and 2015 increased from 960 to 4032, affecting mostly the younger age group [6]. Globalization, trade, travel, demographic trends, and warming temperatures have all contributed to an increase in the global incidence of DHF [7]. In addition, DHF transmission and disease exhibit inherently dynamic spatial and temporal patterns due to a changing environment and population immunological profile [8]. The heterogeneous risk factors for DHF make it difficult to consider epidemiological changes as a single factor. Based on Kahn et al. (2018) [9] stated that the current expansion of dengue appears multifactorial and may include climate change, virus evolution, and societal factors such as rapid urbanization, population growth and development, socioeconomic factors, and global travel and trade. Despite its complexity, analysis of variables related to the distribution of DHF risk can be a useful tool for generating spatial and temporal scenarios of DHF for surveillance [8]. DHF surveillance is useful for the systematic and continuous collection, recording, analysis, interpretation, and dissemination of DHF that reflects the current health status of a community or population so that actions can be taken to prevent or control DHF [10].

Several researchers have developed a model to predict the DHF risk to assist surveillance in determining DHF policy [11]. The output of these models can aid in decision-making processes regarding control purposes and surveillance methods, as well as serve as good predictive tools in the future. Prediction is a component of surveillance and, more specifically, early warning systems [12]. It is the timely collection and analysis of data, as well as the application of risk-based assessments to prompt decision-making processes that trigger disease intervention strategies to minimize the impact on a specific population. The involvement of various factors originating from the human, animal, and insect sectors, as well as the disease itself, makes early warning systems for DHF risk particularly complex [13]. The authors investigate the various models available for DHF surveillance and their use as an early warning tool and the factors that influence DHF risk. The model’s ability to be an effective risk-reduction tool has been used to improve the public health surveillance system in a variety of ways. They may have similar structural designs, functions, and analytical approaches, but they perform and predict DHF outbreak risk differently [14–16]. There is an increasing number of research reports on DHF outbreak prediction tools. Racloz et al. (2012) [12] previously highlighted the benefits of combining various epidemiological tools, such as mapping and mathematical models, to create an early warning system. Another study by Louis et al. (2014) [8] focused on risk-mapping for DHF from 2005 to 2013. Baharom et al. [13] reviewed the most recent literature (2014–2021) and discussed the evidence for various early warning systems, their performance, and ability to predict DHF outbreaks.

The research gap is visible in the scope of DHF risk modelling, particularly from 2011 to 2021, lacks current investigations into DHF dynamics and risk factors. To fill this void, we hypothesized that a thorough and updated examination of predictor variables and modelling techniques during this time period will not only address research gap but also contribute to the improvements of DHF risk maps. This pioneering investigation not only addresses the prevailing research gap but also promises to yield a novel contribution in the form of refining the DHF risk maps, thereby ushering in a new era of data-driven and well-informed public health decision-making. The purpose of this scoping review is to summarize the most recent literature on the risk model of DHF (2011–2021), as well as to compare the most important predictors and the most commonly used modelling methods in order to generate specific types of risk maps with varying applicability and relevance for public health decision making.

## Materials and methods

### Data source and search strategy

The Preferred Reporting Items for Systematic Reviews and Meta-Analyses (PRISMA) 2020 statement is used to report this scoping review. It took place from January to August 2022. Systematic searching strategies include identification, screening, and the eligibility process. In the identification stage, synonyms and variations were used to enrich the keywords, then applied in the search process [13]. The combinations of search terms we use include; (i) “spatial model” and “Dengue risk”; (ii) “risk” and “Dengue” and “spatial analysis”; (iii) “hazard” and “Dengue” and “Geographic Information System” (iv) “vulnerability” and “Dengue” and “Geographic Information System”; (v) “capacity” and “Dengue” and “spatial analysis”; (vi) “Factor influence” and “dengue risk”. The following databases were searched electronically; PubMed, Scopus, Science Direct, and Google scholar in the first step of the multi-level approach we used Medical Subject Heading terms (for PubMed) and undertook plain text searches for keywords connected with Boolean operators. There were no limitations on the time frame or language of publishing. Even though the search was limited to the English language, no articles that were published in other languages were disqualified from full text evaluation [8].

### Inclusion and exclusion criteria

**Fig. 1:**
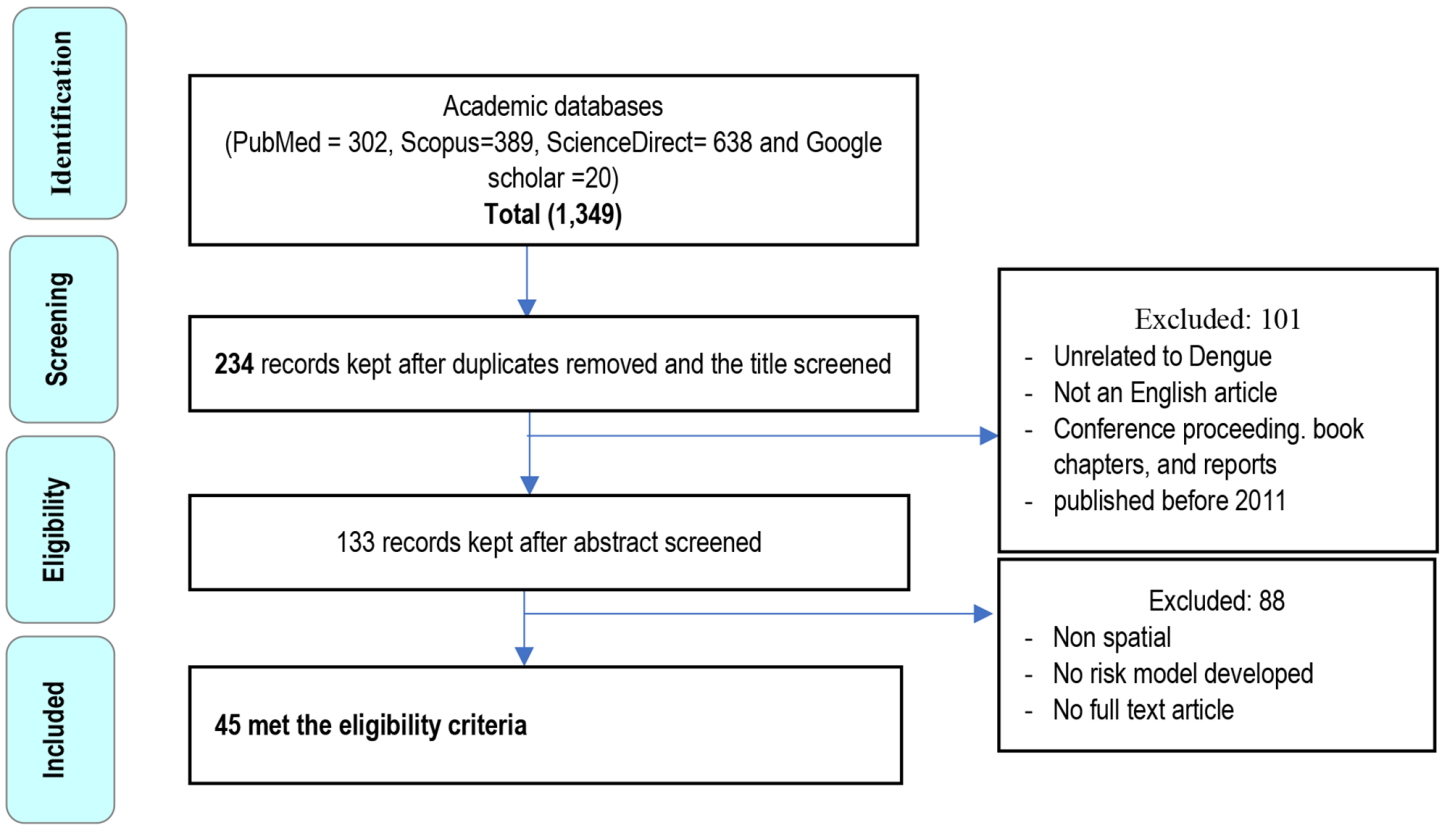
Stages of systematic review

Based on the developed review question and specific inclusion and exclusion criteria, two authors screened the title and abstract. The results were pooled using Mendeley, and duplicates were removed during a second round of revisions. After retrieving the search results, papers for inclusion were selected in two stages. The first step involved two independent researchers selecting articles from the search results based on titles and abstracts, as well as specific inclusion and exclusion criteria. Additional literature was found by scanning the bibliographies of the reviewed articles. The full text of studies related to the research questions was examined. Based on the title of the abstract screen, research whose inclusion appeared ambiguous was included. In the second stage, all articles underwent a full-text review by two reviewers (Fig. 1).

For eligibility, 45 full-text articles were successfully retrieved. All full-text articles were reviewed independently by two authors. All studies that were discovered to be unrelated to the interest and intended outcome were excluded. The reasons for the article’s omission were documented. A number of 88 articles were excluded because they were: (1) non-spatial, (2) did not have a risk model, and (3) did not have a full-text article. The remaining 45 articles were eligible for data extraction (Fig. 1).

### Data extraction

Two author and an independent reviewer performed data extraction using pretested data extraction forms and stored these in a Microsoft Excel 2019 spreadsheet. Disagreements were resolved by consensus. The following publications characteristics was extracted from each paper: publication year, country, region, administrative level (district, municipality, regency/city, province, and country, region, and continental), type of scale (urban or rural), spatial model (Multiple Criteria Decision Making, Statistical, and Machine Learning), dengue data use, and categories of predictors (population, demographic, social-economy, climatology, environmental, entomological, capacity, and epidemiological). The modeling variables are summarized in each study’s narrative description (Table 1).

## Results

### Characteristics of included studies

A comprehensive search of international peer-reviewed journals published from 2011 onwards yielded a total of 1329 published articles. These, 1349 articles were obtained from four databases (302 from PubMed, 389 from Scopus, 638 from Science Direct, and 20 from Google Scholar). Following removal of duplicates and screening of the title 234 articles remained. Of these, 101 articles were excluded for the following reasons unrelated to DHF, not an English article, conference proceedings, book chapters, reports, and published before 2011. A total of 88 articles were then excluded from the remaining 133 articles due to nonspatial, no risk model developed, and full text articles no available. Finally, the systematic review included 45 publications that met the eligibility criteria (Fig. 1; Table 1).

**Fig. 2:**
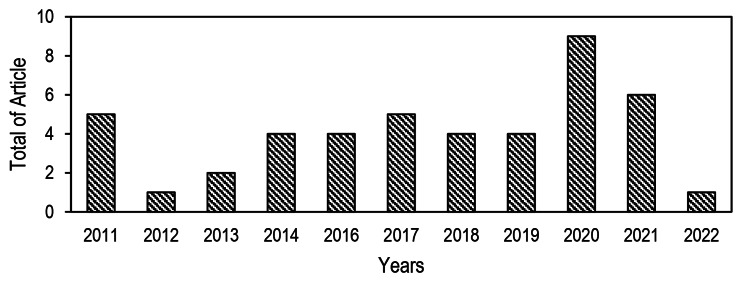
Number of studies based on year of publication from 2011 to 2022

**Fig. 3:**
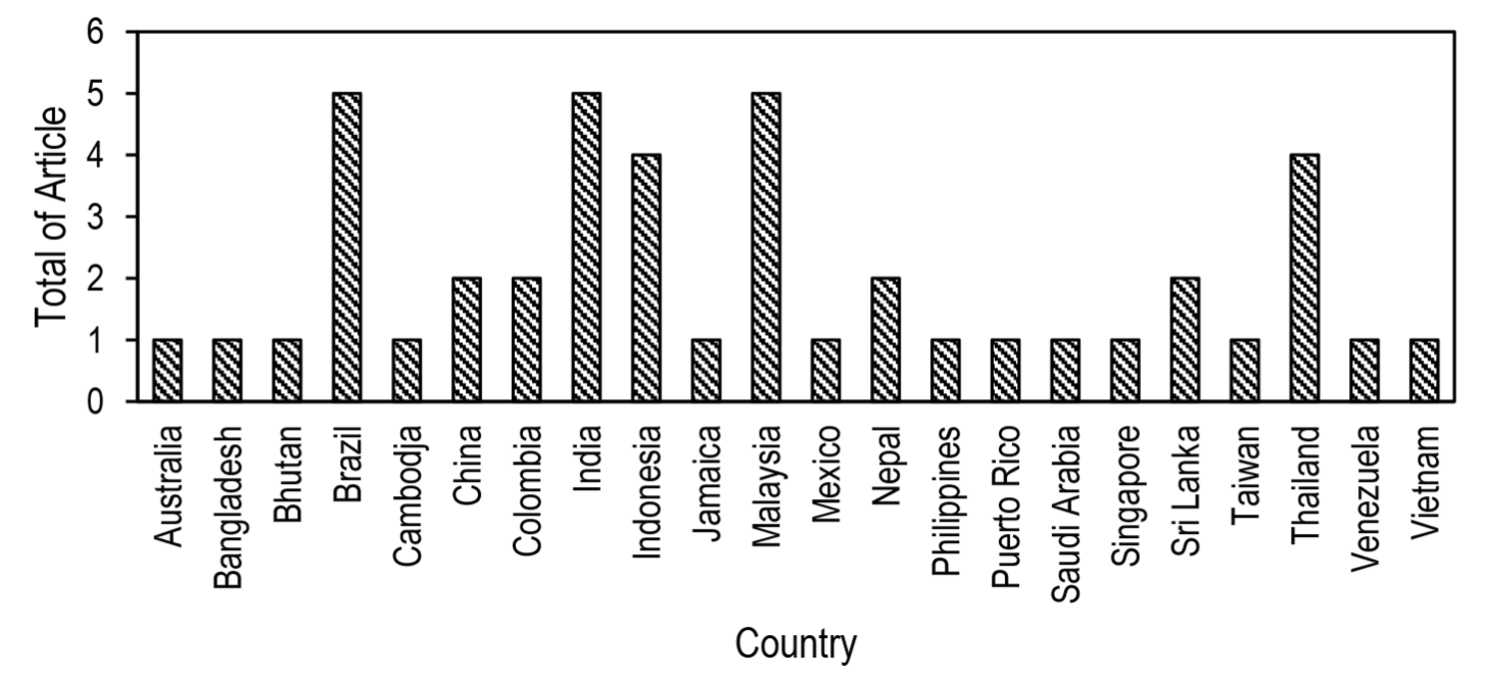
Number of included articles by country of study

Journals published in the last 12 years were used as the deadline for this study (2011–2022). Since 2011, the number of publications has increased. The majority of studies (n = 29, 64.44%) have been published after 2017 (Fig. 2). This systematic review included 45 studies that met the eligibility criteria. Thirty-three (73.3%) of the 45 studies were conducted in Asia, eleven (24.4%) in America, and one (2.2%) in Africa. Half (51.11%) of the studies were conducted in Brazil, India, Indonesia, Malaysia, and Thailand (Fig. 3). The most extended surveillance data time frame was 24 years (33), followed by 18 years (41). Generally, studies used monthly and annual data units. The characteristics of included studies are summarized in Table 1.

### Scale and scope

**Fig. 4:**
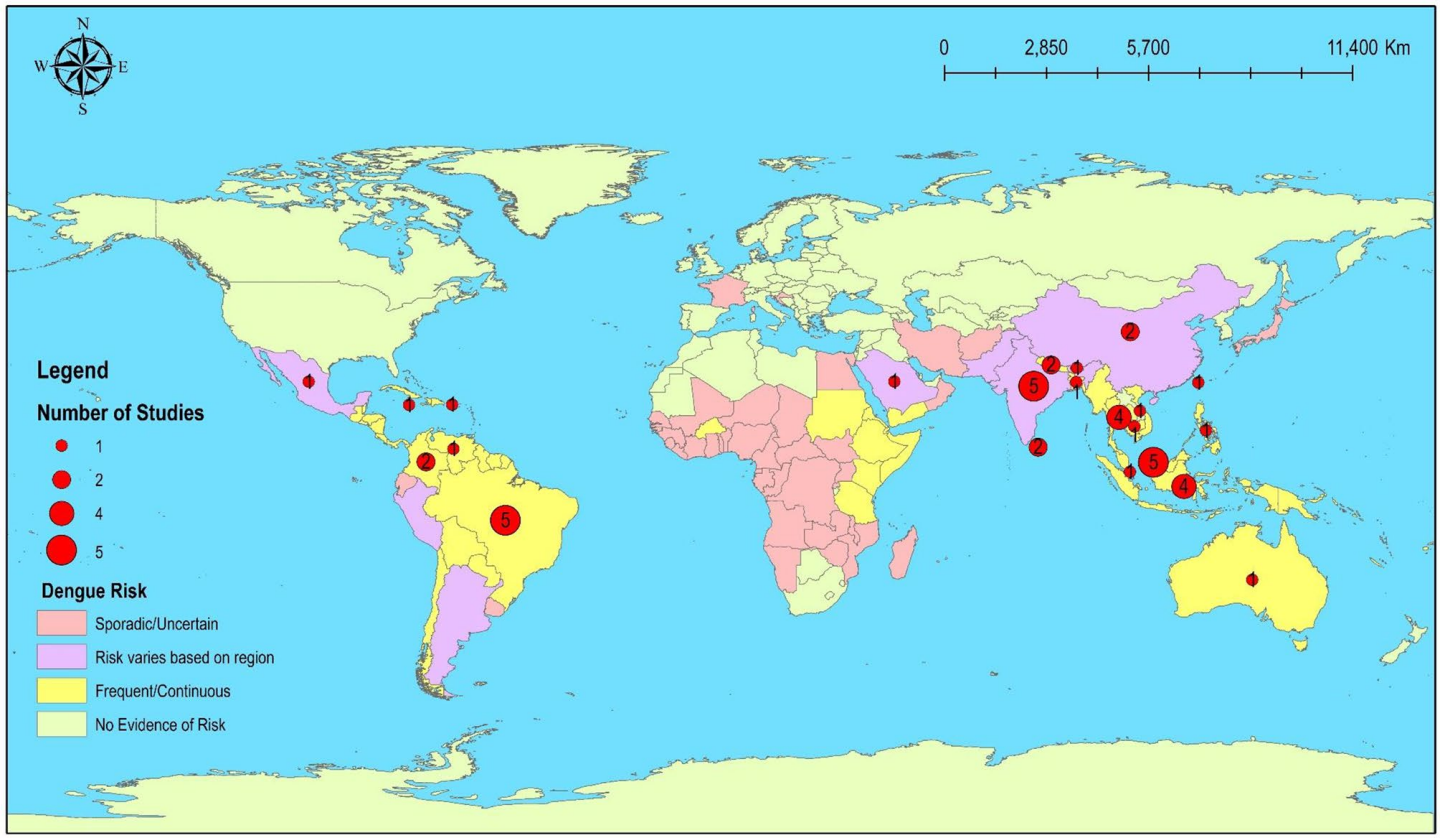
Map of areas with dengue risk based on The Centers for Disease Control and Prevention, The National Center for Emerging and Zoonotic Infectious Diseases (NCEZID), Division of Vector-Borne Diseases (DVBD) with number of publications reviewed in respective countries

DHF risk maps provide localized risk assessments by considering the specific characteristics of an area. By analyzing data at a smaller scale, such as neighborhoods or districts within a city, these maps can highlight areas that are more susceptible to dengue transmission. This information is crucial for local health authorities to implement targeted interventions, allocate resources effectively, the efficiency of control measures and reduces the burden on resources [17]. Of the 45 total studies, 39 (86.67%) were applied to the administrative level, such as district, municipality, regency/city, province, and country, and 6 (13.33%) were applied to the continent and region, respectively. Studies were done at the regency/city, province, country, region, and continental levels were more likely to include a mix of urban and rural areas. Urban and rural (mixing) (62.22%) and rural (28.89%) was the most common study area analyzed (Table 1). Because they were more general and geographically extended, studies done at country, state and province levels were more likely to include rural areas. The distribution of studies by region, country and administrative level is shown in Fig. 4.

### Study design

All studies were conducted retrospectively. Retrospective descriptive is research conducted with the main aim of making a picture or description of a situation objectively by looking back. Although some studies (8, 14, 15, 21, 23, 24, 34, 36, 39, 45) did not use DHF data as a reference, the majority of them used secondary data on DHF cases from health surveillance system surveys. Aside from reported DHF cases, critical predictors for model generation and DHF risk maps included variables from a variety of categories, including population, demography, socioeconomic, climatology, environmental, entomological, capacity, and epidemiological data. The peer-reviewed articles used data ranging from one to twenty years. Eleven studies (1, 5, 6, 9, 10, 11, 16, 18, 28, 32, 37) used data sets with a duration of fewer than three years, while twenty-four of the 45 studies used data sets with a duration of three years or longer (2, 3, 4, 7, 12, 13, 17, 19, 20, 22, 25, 26, 27, 29, 30, 31, 33, 35, 38, 40, 41, 42, 43, 44) (Table 1).

### Predictors

#### Climatology data

Twenty-one studies incorporated climatic predictors in their model generation (1, 2, 6, 7, 9, 13, 16, 22, 26, 27, 29, 31, 32, 33, 34, 35, 36, 39, 40, 41, 43) (Table 1). Three studies used precipitation, temperature, and humidity (26, 29, 35). Predominantly used predictors were precipitation and temperature (2, 6, 7, 9, 16, 22, 27, 31, 33, 34, 39, 40, 41, 43), and eleven studies used only one rain parameter, namely 1, 13, 16, 19, 23, 24, 28, 30, 32, 34, 36. Thirteen studies used climatological stations to interpolate the spatial distribution of temperature and eight studies used remote sensing to analyze Land Surface Temperature (LST) (13, 19, 23, 24, 28, 30, 31, 34). They used remotely sensed data on climatic variables to address the lack of routinely collected data from meteorological stations. These climatological variables were used to characterize the spatial and temporal DHF risk.

#### Environmental data

Environmental information comprised data on elevation, slope, land use/land cover, vegetation, and microenvironment. Elevation and slope derived from Digital Elevation Model (DEM) (1, 14, 16, 24, 27, 28, 32, 42, 43). Extract each studied DEM that represents the various grid-cell sizes used in the worked example. Aedes aegypti are abundant, and endemic DHF virus transmission occurs in low-elevation areas but where a large proportion of the human population lives in high-elevation cities located above the elevation ceiling below which local climates allow for the proliferation of the mosquito vector and endemic DHF virus transmission [18].

Twenty-five studies generated land use/landcover (1, 2, 3, 9, 13, 14, 15, 16, 21, 23, 24, 25, 28, 29, 30, 31, 32, 34, 35, 37, 39, 40, 42, 43, 45). Generally, land use/land cover consists of built-up, vegetation, agriculture, and waterbodies. Developed land has been shown to increase DHF incidence, and urban land has been shown to increase the risk of extended DHF distribution [19]. Vegetation cover was obtained from Enhanced Vegetation Index (EVI) and Normalized Difference Vegetation Index (NDVI). The simultaneous deterioration of vegetation cover (vigor and density) in the area was directly related to an increase in the occurrence of DHF cases [19]. Eleven studies (13, 19, 23, 24, 25, 26, 28, 29, 30, 31, 34) used satellite images to gain information on the environmental vector breeding appropriateness. Enhanced Vegetation Index (EVI) and Normalized Difference Vegetation Index (NDVI) can be used to quantify vegetation greenness, The Normalized Difference Built-up Index (NDBI) uses the NIR and SWIR bands to emphasize manufactured built-up areas, and The Normalized Difference Water Index (NDWI) is known to be strongly related to the plant water content. Three studies (13, 31, 34) were sourced from the Moderate Resolution Imaging Spectroradiometer (MODIS), and eight studies (19, 23, 24, 25, 26, 28, 29, 30) were sourced from Landsat 8 Operational Land Manager and Thermal Infrared Sensor (OLI/TIRS) imagery. Meanwhile, predictors used in microenvironmental data include the following: garbage, and sanitation, (4, 7, 36, 37, 40). One of the key factors influencing DHF in developing countries is a lack of basic sanitation facilities, waste management, and clean water [20].

**Table 1:** Data Extraction

No.	Author (s) /Years	Cite	Publications Characteristics			Type of Scale	DHF Data Use	Predictors					Total
			**Country**	**Region**	**Administrative** **Level**			**Climatology**	**Environmental**	**Entomological**	**Capacity**	**Epidemiological**	
1	Jeefoo & Tripathi (2011)	[21]	Thailand	Asia	Province	U&R	2007	●	●			●	3
2	Shafie, A. (2011)	[22]	Malaysia	Asia	Province	U&R	2000–2004	●	●			●	3
3	Khormi & Kumar (2011)	[23]	Saudi Arabia	Asia	County	U	2006–2010		●			●	2
4	Cordeiro et al. (2011)	[24]	Brazil	Americas	Municipality	U	2006–2007		●	●	●	●	4
5	Schmidt et al. (2011)	[25]	Vietnam	Asia	District	U&R	2005–2007				●	●	2
6	Hu et al. (2012)	[26]	Australia	Asia	Province	U&R	2002–2005	●				●	2
7	Dickin et al. (2013)	[27]	Malaysia	Asia	Country	U&R	2001–2011	●	●		●	●	4
8	Hagenlocher et al(2013)	[28]	Colombia	Americas	City	U	-				●	●	2
9	Dickin & Schuster-Wallace (2014)	[29]	Brazil	Americas	Region	U&R	2010	●	●		●	●	4
10	Pastrana et al. (2014)	[30]	Brazil	Americas	City	U	2009–2011					●	1
11	Barbosa et al. (2014)	[31]	Brazil	Americas	Municipality	U&R	2011			●		●	2
12	Chiu et al. (2014)	[32]	Taiwan	Asia	Regency and District	U&R	2004–2011						1
13	Wijayanti et al. (2016)	[33]	Indonesia	Asia	Regency	U&R	2000–2013	●	●		●	●	4
14	Dom et al. (2016)	[34]	Malaysia	Asia	Province	U&R	-		●			●	2
15	Delmelle et al. (2016)	[35]	Cambodja	Asia	Municipality	U	-		●		●	●	3
16	Attaway et al. (2016)	[36]	Africa	Africa	Continent	U&R	2012	●	●			●	3
17	Dom et al. (2017)	[37]	Malaysia	Asia	District	U	2006–2010					●	1
18	Vincenti-Gonzalez et al. (2017)	[38]	Venezuela	Americas	Municipality	U	2010–2011				●	●	2
19	Martínez-Bello et al. (2017))	[39]	Colombia	Americas	Province	U&R	2008–2015	●	●				2
20	Hafeez et al. (2017)	[40]	India	Asia	District	R	2007–2013					●	1
21	Panhwer et al. (2017)	[41]	India	Asia	Regency	U&R	-		●			●	2
22	Acharya et al. (2018)	[42]	Nepal	Asia	District	R	2011–2016	●					1
23	Acharya et al. (2018)	[43]	Nepal	Asia	Country	U&R	-	●	●			●	3
24	Ajim Ali & Ahmad (2018)	[44]	India	Asia	Municipality	U	-	●	●			●	3
25	Ong et al. (2018)	[45]	Singapore	Asia	Country	U	2006–2016		●			●	2
26	Ordoñez-Sierra et al. (2019)	[46]	Mexico	Americas	Region	U&R	2009–2015	●	●			●	2
27	Dhewantara et al. (2019)	[17]	Indonesia	Asia	Province	U&R	2012–2017	●	●			●	3
28	Ghosh et al. (2019)	[47]	India	Asia	Municipality	U	2017	●	●			●	3
29	Zheng et al. (2019)	[48]	China	Asia	Region	U&R	2010–2014	●	●			●	3
30	Sahdev et al. (2020)	[49]	India	Asia	Municipality	U	2012–2017	●	●			●	3
31	Pham et al. (2020)	[50]	Philippines	Asia	Region	U&R	2001–2016	●	●		●	●	4
32	Hnusuwan et al. (2020)	[51]	Thailand	Asia	Province	U&R	2018	●	●			●	3
33	Puggioni et al. (2020)	[52]	Puerto Rico	Americas	Country	U&R	1990–2014	●				●	2
34	H et al. (2020)	[53]	Jamaica	Americas	Country	U&R	-	●	●		●	●	4
35	Udayanga et al. (2020)	[54]	Sri Lanka	Asia	District	R	2012–2019	●	●	●	●	●	5
36	Souza et al. (2020)	[55]	Brazil	Americas	Municipality	U&R	-	●	●	●	●	●	5
37	Wongpituk et al. (2020)	[56]	Thailand	Asia	Province	U&R	2013–2015		●	●	●	●	4
38	Yajid et al. (2020)	[57]	Malaysia	Asia	District	R	2012–2016					●	1
39	Tsheten et al. (2021)	[58]	Bhutan	Asia	Province	U&R	-	●	●			●	3
40	Zafar et al. (2021)	[59]	Thailand & LAO PDR	Asia	Country	U&R	2003–2019	●	●		●	●	4
41	Riad et al. (2021)	[60]	Bangladesh	Asia	Country	U&R	2000–2018	●				●	2
42	Withanage et al. (2021)	[61]	Sri Lanka	Asia	District	Urban	2014–2018		●	●		●	3
43	Wu et al. (2021)	[62]	China	Asia	Region	U&R	2003–2014	●	●			●	3
44	Faridah et al. (2021)	[63]	Indonesia	Asia	City	Urban	2014–2016		●		●	●	3
45	Pakaya et al. (2022)	[64]	Indonesia	Asia	Regency	U&R	-		●			●	2
							Total	26	32	6	15	42	

#### Entomological data

Entomological data were collected at the egg, larval, and adult stages. Mosquito egg and larval collections were conducted using dark containers containing water and a substrate, traditional dipping techniques, or the recording of an index (Breteau index, container index, and house index). Two studies used the Bretau Index (BI) (35, 37), which defined the number of positive containers/number of houses explored × 100. Three studies used the house index (HI) (35, 36, 37), which is defined as the number of infected houses × 100/total number of houses, and two studies used the Container Index (CI) (37, 42), which define the number of infected containers × 100/total number of containers [65]. Two studies (4, 11) used egg, larval, and adult indicators as input predictors for model generation. Capture activities used different types of traps to estimate adult mosquito population densities.

#### Capacity

Capacity indicators reflect populations’ ability to cope with or prevent DHF outbreaks by assessing their preparedness and response mechanisms [59]. Eleven studies stated women and their education level play critical roles in the home (4, 5, 7, 8, 9, 15, 34, 35, 36, 37, 40). They frequently manage household conditions, waste, water, the family’s health care, etc. Households with a low female literacy rate and education level had an increased risk of Aedes aegypti oviposition sites around them. Good access to healthcare (2, 7, 8, 9, 13, 15, 31, 34, 40, 45) can reduce susceptibility, particularly to complications, and increase early disease diagnosis and immediate medical care to reduce morbidity and thus reduce population susceptibility; thus, distance to a hospital was included as an indicator of adaptive capacity [59]. One study uses mosquito preventive measures (17) like litter outdoors, used car tires outdoors, bottles outdoors, indoor flower vases, screened windows/doors, mosquito nets, insecticide repellent, and container washing. The presence of the used container, litter outdoors, bottles outdoors, indoor flower vases, and used car tires outdoors may create a suitable environment to develop mosquito breeding sites [66]. Mosquito vectors bite continuously throughout the day, these precautions must be taken both inside and outside the home (for example, at work/school), like screened windows/doors, mosquito nets, and insecticide repellent [63]. Three studies use poverty households (31, 36, 40) as a DHF risk because in areas with high poverty households, there is often limited access to basic amenities like clean water, proper sanitation, and healthcare services. These conditions can contribute to the proliferation of Aedes aegypti and increase the DHF risk. Additionally, poverty may lead to overcrowded living conditions, which can facilitate the spread of the virus [59].

#### Epidemiological

Epidemiology is the study of the distribution, patterns, and determinants of health-related events, including diseases, within a population. The majority of studies (1, 2, 3, 5, 7, 8, 9, 13, 14, 15, 16, 23, 24, 27, 29, 31, 32, 34, 35, 39, 40, 43, 45) described and modelled the DHF risk occurrence using population distribution and density (Table 1). These data were derived primarily from the national censuses. Population density data were used in the 23 articles reviewed. The demographic data used several predictors including age, gender, race/ethnicity, occupation, and sex. These were used in 16 of 45 studies reviewed (4, 5, 7, 8, 9, 15, 18, 21, 31, 34, 35, 37, 38, 40, 41, 44). Meanwhile, the predictors used in social and economic data included; housing type, residential buffer, urban proportion, proximity to road/road network, toilet type, mean distance to hospital, poverty incidence, number of household rooms, number of persons per household, crowding, duration of residence, people who did not study nor worked, following any type of study, having a job, household density and gross domestic product (GDP). Most studies that included socio-economic predictors identified some of them as significant.

The majority of DHF cases were caused by infection with one of the four dengue virus serotypes (DENV) (1–4). Seventeen studies (2, 3, 12, 16, 17, 20, 21, 23, 28, 30, 32, 33, 35, 36, 37, 38, 42) used DF/DHF cases or incidence. One study (1) used DF/DHF incidence buffer. DF/DHF is a viral infection spread by mosquitoes, that live for an average of 8–15 days and can fly 30–50 m per day, covering a distance of 240–600 m in their lifetime [21]. One study (10) used the incidence rate. An incidence rate describes how quickly a disease occurs in a population.

#### Modelling approaches

**Fig. 5:**
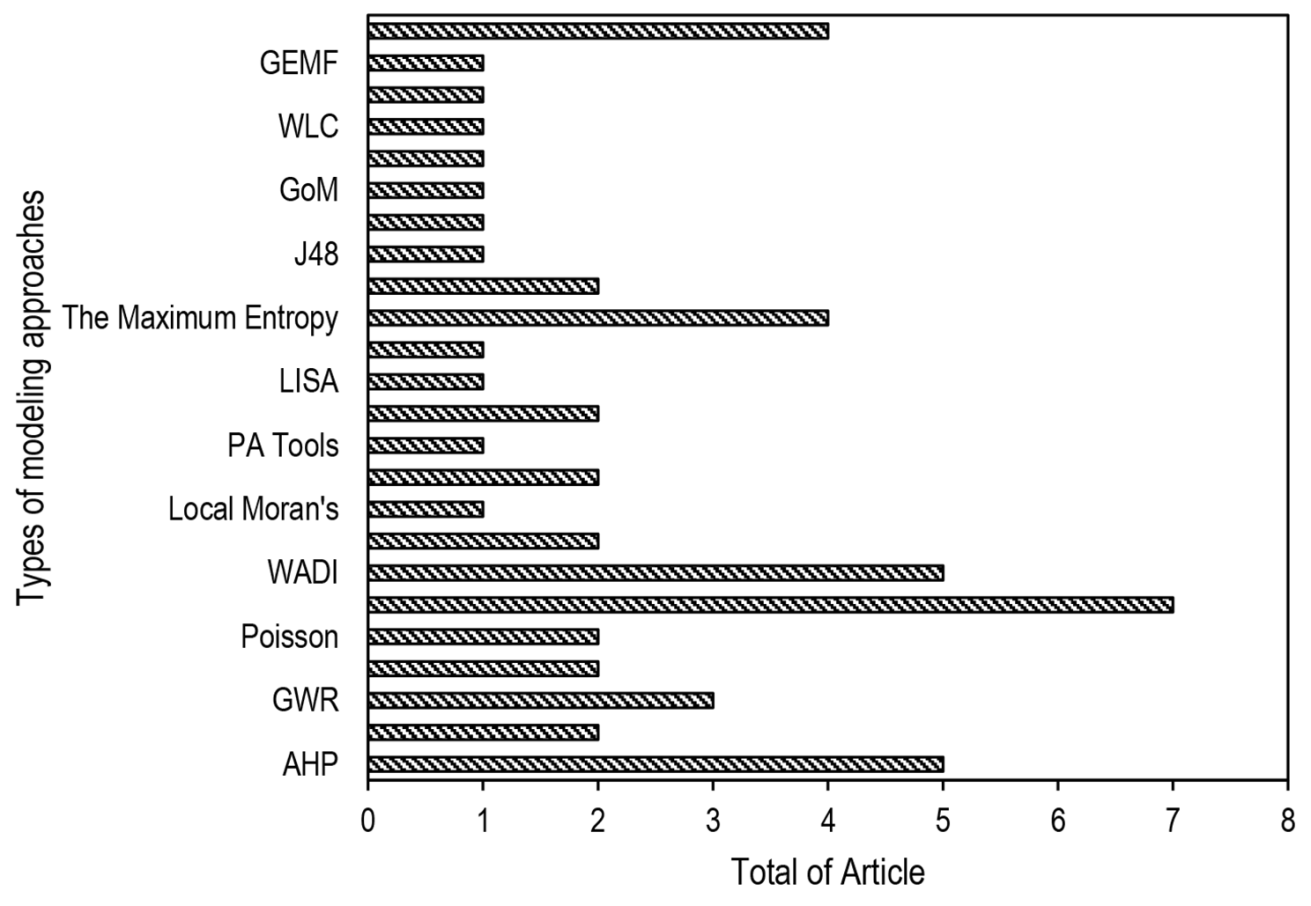
Types of modeling approaches

DHF risk maps were created using a variety of Multi Criteria Decision Making (MCDM), statistical, and Machine Learning (Fig. 5). The majority of studies employed MCDM approaches. To estimate the DHF risk occurrence over a geographical area, reported DHF cases were combined with other selected predictors (climatology, environmental, entomological, capacity, and epidemiological). The maps were created using values calculated from the chosen predictors for each raster map (smallest surface area with a specific value). In terms of methodology, we distinguish the use of models from the use of indices in order to obtain risk estimates. Models imply the use of variables individually, whereas indices use a composite of variables computed from available data.

Seven studies (6, 12, 13, 17,19, 27, 33) used the Bayesian approach. It has the advantage of fully quantifying uncertainty in estimates and can handle small sample sizes [58]. A Bayesian spatial model was used to generate a map of DHF relative risk in an area and investigate the relationship between socio-environmental factors and DHF risk using spatial autocorrelation [17]. Five studies (7, 9, 31, 34, 40) used The Water Associated Disease Index (WADI) to identify and visualize vulnerability to different water-associated diseases by integrating a range of social and biophysical determinants in map format [27, 29, 50, 53, 59]. It aims to assess vulnerability by integrating disease-specific measures of environmental exposure (i.e., temperature, precipitation, land use, etc.) with disease-specific measures of social susceptibility (i.e., life expectancy, educational attainment, access to healthcare, etc.) to provide a holistic picture of risk to disease. The WADI tool complements early warning models for water-associated disease by providing upstream information for planning prevention and control approaches, which increasingly require a comprehensive and geographically broad understanding of vulnerability for implementation [27].

Multi Criteria Decision Making (MCDM) is considered a complex decision-making tool involving quantitative and qualitative factors. Several studies (1, 14, 24, 30, 45) have been found engaging the application of AHP in DHF risk assessment through multi-criteria decision analysis and the use of GIS and high-resolution satellite data to find out DHF risk areas [44]. Furthermore, using AHP with GIS for DHF risk zonation modeling and mapping can display construct results with spatial relationships. Understanding the pattern and distribution of DHF outbreaks can be aided by the spatial relationship. AHP assists decision makers in making correct decisions by putting their intensity of importance, and inconsistency will appear if the decision is incorrect. Maximum Entropy (MaxEnt) is a machine learning program that uses presence-only data to predict distributions based on the principle of maximum entropy [43, 46, 51, 62]. The basic principle of the MaxEnt model is to estimate the potential distribution of DHF by determining the distribution of the maximum entropy (i.e., closest to uniform), with constraints imposed by the observed spatial distributions of the DHF and the environmental conditions (23, 26, 32, 43) [43].

Geographically Weighted Regression (GWR) among others is the most widely used multivariate local statistics to cope with spatially non-stationary processes that allowed to change of parameters locally [23, 35, 42]. GWR can measure the spatial dependency in a dataset and is easily understood due to the traditional regression-based framework. The GWR model was compared to describe the spatial relationship of potential environmental and socioeconomic risk factors with DHF incidence (3, 15, 22). Four studies were conducted to determine the DF incidence hotspot zonation map using the Kernel Density Estimation (KDE) method (10, 17, 42, 44). KDE was used to fit a smoothly tapered surface to point layers, and Euclidean distance was used to identify polygon layer close exposures. The risk values for each layer were ranked based on their contribution to DHF incidence transmission [61]. Two studies used each model i.e., Stepwise Logistic Regression (2, 26), Multinomial Logistic (4, 28), Poisson (5, 42), Principal Component Analysis (8, 35), Generalized Additive Models (11, 29), Logistic Regression (18, 37), Random Forest (25, 32). The Random Forest method is a powerful and widely used machine learning algorithm that can be applied to the creation of a dengue risk map. It is to predict and visualize areas with a higher likelihood of DHF outbreak or transmission. Several models were only used by one study e.g., Local Moran’s, PA Tools; LISA, OLS, J48, GoM, ANN, WLC, BWM, GEMF (Figuer 4).

## Discussion

### Retrospective studies

Most of studies were conducted retrospectively and non-retrospective studies are not the primary choice in dengue risk studies. Non-retrospective studies were not the main choice in dengue fever risk studies due to limitations in search terms, currently there is an increasing number of early warnings, machine learning, and AI modeling in DHF risk that cover location coverage but cannot be extracted through the identification stage. This is due to the combination of search terms used that are closely related to “spatial” and “Geographic Information Systems”, where these two keywords are closely related to retrospective research which involves the collection and analysis of data that has occurred in the time period before the research began. This means the study looks back to collect data and tries to identify relationships between past factors. DHF risk can vary spatially over time. Retrospective studies, which involve collecting historical data over an extended period, allow for the examination of how DHF risk has evolved and fluctuated in different geographic areas (spatial patterns). Additionally, they provide the opportunity to identify and analyze spatial trends in dengue risk factors and help researchers detect hotspots or clusters of dengue cases and investigate the factors contributing to these clusters. The DHF risk is influenced by various factors related to the past, including climate, urbanization, population movements, and vector distribution. Understanding the historical context of these factors is critical to developing accurate spatial models. Retrospective data can provide insight into how these factors change over time.

### Predictors

Different approaches to DHF risk mapping employed a wide range of predictors without following a predictable pattern. This observation highlights the complexity and variability of DHF transmission dynamics and the challenges involved in accurately predicting and mapping DHF risk [67]. It suggests that researchers and practitioners in this field recognized the importance of considering multiple factors and variables to capture the diverse aspects that influence DHF risk. By using a broad set of predictors, researchers likely aimed to capture the complex interactions between climatological, environmental, entomological, capacity, and epidemiological factors that contribute to DHF risk. This approach acknowledges that no single predictor may provide a complete understanding of DHF risk, and a comprehensive assessment requires considering multiple factors simultaneously. In summary, the absence of a pattern of predictor use associated with specific approaches in creating DHF risk maps indicates the adoption of diverse predictors to account for the multifaceted nature of DHF transmission dynamics and create more comprehensive risk assessments.

#### Temperature

DHF risk is influenced by temperature as it affects both the mosquito vector and the virus. Higher temperatures can accelerate the development of the DHF virus within mosquitoes, leading to increased viral replication and a shorter incubation period. Warmer temperatures can also speed up the mosquito’s life cycle, resulting in more frequent breeding and shorter intervals between generations, thereby increasing the chances of DHF risk [68]. **Rainfall**: DHF risk is closely linked to rainfall patterns. Mosquitoes require water for breeding, and rainfall provides ideal breeding sites for the Aedes aegypti. Heavy rainfall can create stagnant water pools and increase the availability of breeding sites. However, excessive rainfall can also flush away breeding sites or dilute larval habitats, reducing mosquito populations. On the other hand, periods of drought followed by rainfall can create temporary water sources, which can lead to a sudden increase in mosquito populations and DHF transmission [69]. **Humidity**: High humidity can facilitate the survival and reproduction of mosquitoes. Aedes aegypti thrive in humid environments, and higher humidity levels can prolong their lifespan and increase their ability to transmit DHF. Additionally, increased humidity can also affect the development of the DHF virus within mosquitoes, potentially leading to higher viral loads and increased transmission [70].

#### Elevation

Generally, the DHF risk is higher in areas with lower elevations. This is because Aedes aegypti, which are responsible for spreading the DHF virus, prefer warm and humid environments. They thrive in areas with stagnant water, such as water containers, flower pots, and discarded tires, which are common breeding grounds for mosquitoes. Lower elevations tend to have higher temperatures and higher humidity, creating more favorable conditions for mosquito breeding and the survival and replication of the DHF virus [71]. Additionally, lower elevations often have a denser population and urban areas, which can further increase the DHF risk due to a higher concentration of potential mosquito breeding sites. In contrast, higher elevations, especially in mountainous or cooler regions, tend to have a lower DHF risk. The cooler temperatures and reduced humidity at higher elevations are less favorable for the survival and reproduction of Aedes aegypti. As a result, the mosquito population may be lower, and the DHF risk decreases.

#### Vegetation and land-use

The presence of dense vegetation and specific land-use types can influence the DHF risk. Dense vegetation provides shaded areas and increased moisture, creating suitable resting and breeding sites for mosquitoes. Dense vegetation with thick foliage can provide shade and retain water, creating favorable conditions for mosquito breeding [72]. Overgrown vegetation can also make it difficult to detect and eliminate mosquito breeding sites. Vegetation plays a crucial role in maintaining ecological balance and regulating mosquito populations. In healthy ecosystems, various predators, such as dragonflies, birds, and bats, help control mosquito populations. Vegetation can contribute to local climate regulation by providing shade and reducing temperature [73]. Changes in vegetation cover can alter local microclimates [74], potentially affecting mosquito populations and DHF transmission dynamics. Certain land-use types, such as urban areas with inadequate waste management systems, can contribute to the accumulation of water in containers and other objects, creating ideal breeding grounds for mosquitoes. Rapid urbanization often leads to the creation of more artificial containers and increased water storage practices, providing additional breeding sites for mosquitoes [75].

**Capacity.** The presence of well-equipped healthcare facilities, including hospitals and clinics, plays a crucial role in diagnosing and treating DHF cases promptly. A region with limited healthcare infrastructure may face challenges in identifying and managing DHF cases effectively, potentially leading to increased transmission and more severe outcomes. Regions with strong vector control programs, such as regular monitoring, surveillance, and effective mosquito control strategies, can significantly reduce the mosquito population and minimize the DHF risk. Adequate resources and infrastructure for implementing such measures are essential for mitigating the risk. Timely and accurate surveillance systems are vital for detecting DHF cases, monitoring disease trends, and implementing appropriate control measures. Regions with robust public health surveillance systems can identify outbreaks early on, allowing for swift intervention and prevention efforts [40]. Educating the public about DHF prevention measures, such as eliminating breeding sites and practicing personal protection methods, can help reduce the DHF risk. Regions with effective community engagement programs and resources for public education are more likely to have a higher level of awareness and adherence to preventive measures [76].

#### Entomological

Understanding the dynamics of mosquito populations and their behavior is crucial for assessing the DHF risk. Entomological measurements provide insights into the abundance and activity of mosquitoes in a given area, which can help predict and monitor the potential for DHF outbreaks [77]. Larvae abundance refers to the number of mosquito larvae found in breeding sites, such as water containers or stagnant water sources [78]. Monitoring larvae abundance helps identify areas with high mosquito breeding activity and serves as an indicator of potential mosquito populations. While these entomological measurements provide valuable data for understanding mosquito populations and activity, their direct relationship to clinical DHF cases is complex and not fully elucidated.

#### Epidemiological

Epidemiology is concerned with studying the distribution and determinants of diseases within populations. The size and density of the population in an area can affect the DHF risk. **Population**. Higher population densities increase the likelihood of DHF transmission by providing more opportunities for mosquitos to come into contact with humans. Furthermore, if infected individuals move between different areas, large populations can facilitate the virus’s rapid spread [67]. **Demographic**. Demographic data is critical in understanding population dynamics and identifying areas that are more vulnerable to DHF outbreaks when developing DHF risk maps at the local level [68]. Age is an important factor, as older individuals may have developed immunity through previous exposure to the DHF, while younger populations, including children, maybe more at risk [69]. **Social-economic**. Social-economic conditions can significantly impact the DHF risk. Poverty and inadequate housing conditions, such as lack of proper sanitation and access to clean water, can contribute to the proliferation of mosquito breeding sites [70]. These conditions often exist in urban slums or overcrowded areas where stagnant water collects and provides ideal breeding grounds for mosquitoes [71]. Lack of resources and infrastructure for mosquito control and prevention measures can also increase the risk. Additionally, socioeconomic factors can impact healthcare-seeking behavior, which may delay the diagnosis and treatment of DHF cases [72, 73]. **DHF Cases**. Epidemiological data on DHF cases, can help public health authorities implement targeted interventions, such as mosquito control measures and public awareness campaigns, to reduce the DHF risk. DHF cases is particularly prevalent in the Asian region [1]. This predominance can be linked to a number of causes, including the region’s tropical and subtropical climate, which offers an ideal setting for the Aedes mosquitos responsible for viral transmission [79]. The high population density in many Asian countries, increasing urbanization, and inadequate sanitary facilities all contribute to the growth of mosquito breeding grounds. Furthermore, in some locations, poor healthcare access and inadequate public health measures may result in underreporting and difficulty in limiting disease spread.

### The weakness of current DHF risk maps

#### Host serological profile and virus genetic diversity

Dengue is caused by four distinct serotypes of the dengue virus (DENV-1, DENV-2, DENV-3, and DENV-4) [80]. Once a person is infected with one serotype, they develop lifelong immunity to that specific serotype but remain susceptible to the other serotypes. This creates a complex pattern of immunity in the population, known as the host serological profile. The immunity levels to different serotypes vary across different geographic regions, as well as within different age groups within the same region. Current DHF risk maps often rely on data such as reported DHF cases and mosquito surveillance to predict the DHF risk. However, these maps may not accurately capture the host serological profile of the population. If a prediction model fails to consider the serological profile and immunity levels in a given area, it may underestimate or overestimate the DHF risk. This can lead to ineffective allocation of resources and public health interventions.

DHF viruses are genetically diverse, even within each serotype. Genetic variations in the virus can influence their ability to transmit and cause severe disease. Some strains may be more virulent, while others may be less likely to cause severe symptoms. Additionally, the genetic diversity of the virus can affect the effectiveness of diagnostic tests and vaccines. Current DHF risk models often do not account for the genetic diversity of the DHF virus. They may assume a uniform distribution of virus strains or rely on limited genetic data. As a result, these models may overlook important variations in viral strains that can impact the transmission dynamics and severity of DHF outbreaks. Consequently, the accuracy of predictions may be compromised.

To address these weaknesses, ongoing research is focused on improving DHF risk models by incorporating data on host serological profiles and virus genetic diversity. By integrating information on population immunity levels and the genetic characteristics of circulating virus strains, it is possible to enhance the accuracy and effectiveness of DHF risk maps.

#### Climate Change

It can have a significant impact on the DHF risk. Rising temperatures associated with climate change create favorable conditions for the Aedes aegypti to thrive [81–84]. Warmer temperatures increase mosquito reproduction rates, shorten the time it takes for mosquitoes to become infectious, and speed up the virus replication within the mosquitoes. As a result, higher temperatures enhance the transmission of DHF virus and can lead to more frequent outbreaks [71]. It can alter rainfall patterns, leading to increased rainfall intensity and changes in the distribution and frequency of precipitation. Heavy rainfall events can create breeding sites for mosquitoes by providing them with more stagnant water, such as in puddles, containers, or water storage facilities. This facilitates the proliferation of Aedes aegypti and increases the chances of DHF transmission [85]. Climate change is also linked to an increase in the frequency and intensity of extreme weather events, such as hurricanes, cyclones, and floods [86]. These events can disrupt sanitation systems, damage infrastructure, and displace populations, leading to the creation of temporary water storage sites. These sites often become breeding grounds for mosquitoes and contribute to the spread of DHF. Changes in climate can also impact the geographic range of the Aedes aegypti, enabling them to expand their range to higher altitudes and latitudes [87, 88]. As a result, areas that were previously unaffected by DHF may become suitable habitats for mosquito breeding and DHF transmission. This expansion exposes populations with little or no prior immunity to the disease, making them more vulnerable. Climate change can disrupt ecological systems and ecological interactions, including those between mosquitoes, their predators, and competitors [89]. These disruptions can affect the natural balance that helps control mosquito populations and limit the spread of diseases like DHF.

Overall, climate change acts as a catalyst for DHF transmission by creating favorable environmental conditions for mosquito breeding, altering mosquito distribution, and influencing the dynamics of DHF virus replication within mosquitoes. Understanding and addressing the link between climate change and DHF risk is crucial for implementing effective preventive measures and public health strategies to combat the disease.

#### Mobility

*Travel and Migration*: People travel between regions, countries, and even continents, potentially carrying the DHF virus with them. This movement can introduce the virus to new areas or contribute to the spread of existing outbreaks [90]. Prediction maps may not account for the movement patterns of individuals, making it difficult to accurately predict the introduction or spread of DHF in different locations. *Urbanization and Urban Mobility*: Rapid urbanization and increased urban mobility play a significant role in DHF transmission. Urban areas provide favorable breeding grounds for mosquitoes, and people frequently move within and between cities, potentially spreading the virus [91]. The movement of infected individuals from one urban center to another can result in the dispersion of DHF cases, challenging the predictive accuracy of static maps. *Seasonal and Temporary Migration*: Seasonal migration, particularly in agricultural or tourist regions, can contribute to the spread of DHF. Temporary workers or tourists moving into DHF-endemic areas may bring the virus from their place of origin or contract the infection locally and then spread it upon returning home [9, 92]. Prediction maps might not account for these temporary movements, leading to inaccurate estimations of DHF risk.

For future studies, DHF risk efforts may need to integrate more dynamic data sources, such as real-time mobility data, social media trends, and other relevant indicators. By incorporating these factors, predictive models can potentially improve their ability to account for the impact of mobility on DHF transmission and produce more reliable risk maps.

#### Scale and temporal resolution

The weakness of current DHF risk maps originating from scale refers to limitations in the spatial resolution or granularity at which the predictions are made [93]. DHF is a vector-borne disease transmitted by Aedes aegypti, and its incidence can vary significantly at different geographic scales, such as district, municipality, regency/city, province, country, region, and continental [8]. At larger scales, such as country, region or continental levels, DHF risk maps may provide a broad overview of the disease risk in a particular area. However, these maps often fail to capture the local heterogeneity within that region. The incidence of DHF can vary widely even within a single city or regency, with certain neighborhoods or communities being more susceptible to outbreaks due to factors like population density, environmental conditions, and socioeconomic factors. Predictions made at a larger scale may not capture these fine-grained variations.

On the other hand, when DHF risk maps are created at a smaller scale, such as at the neighborhood or household level, they may provide more localized information and be useful for targeted interventions. However, generating accurate predictions at such fine scales can be challenging due to limitations in data availability and the complexity of the disease dynamics. Another aspect related to scale is the temporal resolution of the predictions. DHF transmission patterns can change over time, with seasonal variations and fluctuations in mosquito populations [94]. Predictions made at coarse temporal scales, such as yearly or quarterly, may not capture the short-term dynamics and fail to provide timely information for public health interventions.

To overcome the weaknesses of current DHF risk maps originating from scale, it is important to integrate data from multiple sources and use advanced modeling techniques. This includes incorporating high-resolution spatial data, such as satellite imagery or geospatial data on human mobility, and leveraging machine learning or statistical models that can handle complex and dynamic interactions. Furthermore, efforts should be made to collect and analyze local data at smaller scales to improve the accuracy and granularity of the predictions.

#### Limitation in DHF risk modelling

*Data Limitations*: The accuracy and reliability of prediction models heavily depend on the quality and quantity of data available for training [95]. However, there may be limitations in data availability, completeness, or accuracy, which can lead to less reliable predictions. *Complex Interactions*: Predictive models attempt to capture these complex interactions, but it can be challenging to accurately represent all the variables and their relationships [96]. Oversimplification or incomplete understanding of these interactions can result in less accurate predictions. *Uncertainty and Variability*: DHF transmission is subject to inherent uncertainty and variability [84]. Factors such as climate variability, vector control measures, and human mobility can introduce unpredictability in the spread of the disease. Prediction models may struggle to accurately capture these uncertainties and provide precise estimates of DHF risk. *Lack of Incorporating Real-time Data*: Many DHF risk models rely on historical data and static variables. However, real-time data, such as updated mosquito surveillance data or information on ongoing control measures, can significantly enhance the accuracy of predictions. Models that do not incorporate real-time data may fail to capture dynamic changes in DHF transmission patterns. *Model Validation and Generalization*: Predictive models need to be validated and tested against independent datasets to assess their accuracy and generalizability. Inadequate validation or overfitting to training data can lead to models that perform well on historical data but fail to accurately predict future DHF outbreaks.

It is necessary to conduct ongoing research and make improvements to DHF risk models to address these flaws. Incorporating more comprehensive and high-quality data, considering complex interactions, improving spatial and temporal resolution, accounting for uncertainty, integrating real-time data, and thorough model validation are essential steps toward enhancing the accuracy and reliability of DHF risk maps.

## Conclusions

Spatial models of DHF risk offer valuable insights into the distribution and factors influencing the occurrence of DHF. These models utilize geographical, environmental, and epidemiological information to predict high-risk areas, thus, identifying regions where DHF outbreaks are likely to occur. The findings suggest that proactive surveillance and public health actions should focus on high-risk areas identified by these models to effectively control DHF spread and reduce its public health impact. The effectiveness of the spatial DHF risk model has certain limitations. It heavily depends on geographical and environmental aspects such as temperature, precipitation, and vegetation, but fails to account for human behavior and social factors contributing to DHF transmission. Furthermore, the model assumes a static relationship between the environmental factors and DHF risk, overlooking the dynamic nature of the disease amidst changing urban landscapes and population movements. Its accuracy may also suffer due to insufficient data quality in resource-constrained areas, potentially introducing biases and uncertainties into the predictions. Thus, while the Spatial Model offers valuable insights, it oversimplifies the intricate interplay between environmental, social, and human factors in the dynamics of DHF transmission.

### Electronic supplementary material

Below is the link to the electronic supplementary material.

Supplementary Material 1

## Data Availability

This published article and its supplementary information files include all data generated or analyzed during this study.

## References

[1] Sharma H (2022). Does COVID-19 lockdowns have impacted on global dengue burden? A special focus to India. BMC Public Health.

[2] Acharya BK, Cao C, Xu M, Khanal L, Naeem S, Pandit S. Present and future of dengue Fever in Nepal: mapping climatic suitability by ecological niche model. Int J Environ Res Public Health. 2018;15(2). 10.3390/ijerph15020187. 10.3390/ijerph15020187 PMC5857046 29360797

[3] Bhatt S (2013). The global distribution and burden of dengue. Nature.

[4] Ahmad S, Asif M, Talib R, Adeel M, Yasir M, Chaudary MH (2018). Surveillance of intensity level and geographical spreading of dengue outbreak among males and females in Punjab, Pakistan: a case study of 2011. J Infect Public Health.

[5] Goldhardt R, Patel H, Davis JL. “Acute Posterior Multifocal Placoid Pigment Epitheliopathy Following Dengue Fever: A New Association for an Old Disease.,” *Ocul. Immunol. Inflamm*, vol. 24, no. 6, pp. 610–614, Dec. 2016, 10.3109/09273948.2015.1125513. 10.3109/09273948.2015.1125513 26902823

[6] WHO., *Dengue and severe dengue*. 2022.

[7] Ebi KL, Nealon J (2016). Dengue in a changing climate. Environ Res.

[8] Louis VR (2014). Modeling tools for dengue risk mapping - a systematic review. Int J Health Geogr.

[9] Khan J, Khan I, Ghaffar A, Khalid B (2018). Epidemiological trends and risk factors associated with dengue Disease in Pakistan (1980–2014): a systematic literature search and analysis. BMC Public Health.

[10] Runge-Ranzinger S, McCall PJ, Kroeger A, Horstick O (2014). Dengue Disease surveillance: an updated systematic literature review. Trop Med Int Heal.

[11] Siriyasatien P, Phumee A, Ongruk P, Jampachaisri K, Kesorn K (2016). Analysis of significant factors for dengue Fever incidence prediction. BMC Bioinformatics.

[12] Racloz V, Ramsey R, Tong S, Hu W. Surveillance of dengue Fever virus: a review of epidemiological models and early warning systems. PLoS Negl Trop Dis. 2012;6(5). 10.1371/journal.pntd.0001648. 10.1371/journal.pntd.0001648 PMC3358322 22629476

[13] Baharom M, Ahmad N, Hod R, Manaf MRA. “Dengue Early Warning System as Outbreak Prediction Tool: A Systematic Review,” *Risk Manag. Healthc. Policy*, vol. 15, no. April, pp. 871–886, 2022, 10.2147/RMHP.S361106. 10.2147/RMHP.S361106 PMC9078425 35535237

[14] Chumpu R, Khamsemanan N, Nattee C (2019). The association between dengue incidences and provincial-level weather variables in Thailand from 2001 to 2014. PLoS ONE.

[15] Jiang Y, Zhu G, Lin L (2017). Research of dengue Fever prediction in san juan, puerto Rico based on a KNN regression model. Lect Notes Comput Sci (Including Subser Lect Notes Artif Intell Lect Notes Bioinformatics).

[16] Chuang TW, Chaves LF, Chen PJ (2017). Effects of local and regional climatic fluctuations on dengue outbreaks in southern Taiwan. PLoS ONE.

[17] Dhewantara PW (2019). Spatial and temporal variation of dengue incidence in the island of Bali, Indonesia: an ecological study. Travel Med Infect Dis.

[18] Lozano-Fuentes S (2012). The dengue virus mosquito vector aedes aegypti at high elevation in México. Am J Trop Med Hyg.

[19] Gao P (2021). Land use and land cover change and its impacts on dengue dynamics in China: a systematic review. PLoS Negl Trop Dis.

[20] Respati T, Raksanagara R, Wangsaputra A (2020). Basic sanitation: is it an important factor in dengue transmission?. Medical Technology and Enviromental Health.

[21] Jeefoo P, Tripathi NK (2011). Dengue risk zone index (DRZI) for mapping dengue risk areas. Int J Geoinformatics.

[22] Shafie A (2011). Evaluation of the spatial risk factors for high incidence of Dengue Fever and Dengue Hemorrhagic Fever using GIS application. Sains Malaysiana.

[23] Khormi HM, Kumar L (2011). Modeling dengue Fever risk based on socioeconomic parameters, nationality and age groups: GIS and remote sensing based case study. Sci Total Environ.

[24] Cordeiro R, et al. Spatial distribution of the risk of dengue Fever in southeast Brazil, 2006–2007. BMC Public Health. 2011;11. 10.1186/1471-2458-11-355. 10.1186/1471-2458-11-355 PMC3128013 21599980

[25] Schmidt WP (2011). Population density, water supply, and the risk of dengue Fever in Vietnam: Cohort study and spatial analysis. PLoS Med.

[26] Hu W, Clements A, Tong S, Williams G, Mengersen K (2012). Spatial patterns and socioecological drivers of dengue Fever transmission in Queensland, Australia. Environ Health Perspect.

[27] Dickin SK, Schuster-Wallace CJ, Elliott SJ (2013). Developing a vulnerability mapping methodology: applying the Water-Associated Disease Index to Dengue in Malaysia. PLoS ONE.

[28] Hagenlocher M, Delmelle E, Casas I, Kienberger S (2013). Assessing socioeconomic vulnerability to dengue Fever in Cali, Colombia: statistical vs expert-based modeling. Int J Health Geogr.

[29] Dickin SK, Schuster-Wallace CJ (2014). Assessing changing vulnerability to dengue in northeastern Brazil using a water-associated Disease index approach. Glob Environ Chang.

[30] Pastrana MEO, Brito RL, Nicolino RR, de Oliveira CSF, Haddad JPA (2014). Spatial and statistical methodologies to determine the distribution of dengue in Brazilian municipalities and relate incidence with the health vulnerability index. Spat Spatiotemporal Epidemiol.

[31] Barbosa GL, et al. Spatial distribution of the risk of Dengue and the Entomological indicators in Sumaré, State of São Paulo, Brazil. PLoS Negl Trop Dis. 2014;8(5). 10.1371/journal.pntd.0002873. 10.1371/journal.pntd.0002873 PMC4022465 24831806

[32] Chiu CH, Wen TH, Chien LC, Yu HL (2014). A probabilistic spatial dengue Fever risk assessment by a threshold-based-quantile regression method. PLoS ONE.

[33] Wijayanti SPM (2016). The importance of Socio-Economic Versus Environmental Risk factors for reported dengue cases in Java, Indonesia. PLoS Negl Trop Dis.

[34] Dom NC, Ahmad AH, Latif ZA, Ismail R (2016). Application of geographical information system-based analytical hierarchy process as a tool for dengue risk assessment. Asian Pac J Trop Dis.

[35] Delmelle E, Hagenlocher M, Kienberger S, Casas I (2016). A spatial model of socioeconomic and environmental determinants of dengue Fever in Cali, Colombia. Acta Trop.

[36] Attaway DF, Jacobsen KH, Falconer A, Manca G, Waters NM (2016). Risk analysis for dengue suitability in Africa using the ArcGIS predictive analysis tools (PA tools). Acta Trop.

[37] Dom NC, Ahmad AH, Latif ZA, Ismail R (2017). Integration of GIS-based model with epidemiological data as a tool for dengue surveillance. EnvironmentAsia.

[38] Vincenti-Gonzalez MF (2017). Spatial analysis of Dengue Seroprevalence and modeling of transmission risk factors in a Dengue Hyperendemic City of Venezuela. PLoS Negl Trop Dis.

[39] Martínez-Bello DA, López-Quílez A, Torres Prieto A (2017). Relative risk estimation of dengue Disease at small spatial scale. Int J Health Geogr.

[40] Hafeez S, Amin M, Munir BA (2017). Spatial mapping of temporal risk to improve prevention measures: a case study of dengue epidemic in Lahore. Spat Spatiotemporal Epidemiol.

[41] Panhwer MA, Pirzada N, Khahro SH (2017). Spatial risk mapping for Dengue Fever using GIS: a case study of Hyderabad. Sindh Univ Res J.

[42] Acharya BK, Cao CX, Lakes T, Chen W, Naeem S, Pandit S (2018). Modeling the spatially varying risk factors of dengue Fever in Jhapa district, Nepal, using the semi-parametric geographically weighted regression model. Int J Biometeorol.

[43] Acharya BK, Cao C, Xu M, Khanal L, Naeem S, Pandit S (2018). Present and future of dengue Fever in Nepal: mapping climatic suitability by ecological niche model. Int J Environ Res Public Health.

[44] Ajim Ali S, Ahmad A (2018). Using analytic hierarchy process with GIS for dengue risk mapping in Kolkata Municipal Corporation, West Bengal, India. Spat Inf Res.

[45] Ong J (2018). Mapping dengue risk in Singapore using Random Forest. PLoS Negl Trop Dis.

[46] Ordoñez-Sierra R (2019). Spatial risk distribution of Dengue based on the ecological niche model of Aedes aegypti (Diptera: Culicidae) in the Central Mexican Highlands. J Med Entomol.

[47] Ghosh S, Dinda S, Chatterjee D, Das K, Mahata R (2019). The spatial clustering of dengue Disease and risk susceptibility mapping: an approach towards sustainable health management in Kharagpur city, India. Spat Inf Res.

[48] Zheng L, Ren HY, Shi RH, Lu L. “Spatiotemporal characteristics and primary influencing factors of typical dengue fever epidemics in China,” *Infect. Dis. Poverty*, vol. 8, no. 24, pp. 1–12, Mar. 2019, 10.1186/s40249-019-0533-9. 10.1186/s40249-019-0533-9 PMC6440137 30922405

[49] Sahdev S, Kumar M (2020). Identification and mapping of dengue epidemics using gisbased multi-criteria decision making. The case of Delhi, India. J Settlements Spat Plan.

[50] Pham NTT, Nguyen CT, Vu HH (2020). Assessing and modelling vulnerability to dengue in the Mekong Delta of Vietnam by geospatial and time-series approaches. Environ Res.

[51] Hnusuwan B, Kajornkasirat S, Puttinaovarat S (2020).

[52] Puggioni G, Couret J, Serman E, Akanda AS, Ginsberg HS. Spatiotemporal modeling of dengue Fever risk in Puerto Rico. Spat Spatiotemporal Epidemiol. 2020;35. 10.1016/j.sste.2020.100375. 10.1016/j.sste.2020.100375 33138945

[53] Henry S, de Mendonça F (2020). Past, present, and future vulnerability to dengue in Jamaica: a spatial analysis of monthly variations. Int J Environ Res Public Health.

[54] Udayanga L, Gunathilaka N, Iqbal MCM, Abeyewickreme W (2020). Climate change induced vulnerability and adaption for dengue incidence in Colombo and Kandy districts: the detailed investigation in Sri Lanka. Infect Dis Poverty.

[55] Souza MLA, Andrade LMB, Spyrides MHC, Tinoco ICM (2020). Profile eestimates for the analysis of climatic and socio-sanitary vulnerability to dengue in municipalities in Northeast Brazil. Urban Clim.

[56] Wongpituk K, Kalayanarooj S, Nithikathkul C. Geospatial analysis of DHF surveillance model in Si Sa Ket Province, Thailand using geographic information system. Int J Geoinform. 2020;16(3):1–8. Available: https://journals.sfu.ca/ijg/index.php/journal/article/view/1785/899

[57] Yajid MZM, Che Dom N, Camalxaman SN, Nasir RA (2020). Spatial-temporal analysis for identification of dengue risk area in Melaka Tengah district. Geocarto Int.

[58] Tsheten T, Clements AA, Gray DJ, Wangdi K (2021). Dengue risk assessment using multicriteria decision analysis: a case study of Bhutan. PLoS Negl Trop Dis.

[59] Zafar S (2021). Development and comparison of dengue vulnerability indices using gis-based multi‐criteria decision analysis in Lao pdr and Thailand. Int J Environ Res Public Health.

[60] Riad MH, Cohnstaedt LW, Scoglio CM (2021). Risk assessment of dengue transmission in Bangladesh using a spatiotemporal network model and climate data. Am J Trop Med Hyg.

[61] Withanage GP, Gunawardana M, Viswakula SD, Samaraweera K, Gunawardena NS, Hapugoda MD (2021). Multivariate spatio-temporal approach to identify vulnerable localities in dengue risk areas using Geographic Information System (GIS). Sci Rep.

[62] Wu W, Ren H, Lu L (2021). Increasingly expanded future risk of dengue Fever in the Pearl River Delta, China. PLoS Negl Trop Dis.

[63] Pakaya R, Hano YH, Olii MR (2022). Dengue hemorrhagic Fever vulnerability assessment in Gorontalo Regency using analytic hierarchy process and geoinformation techniques. Int J Public Heal Sci.

[64] Faridah L (2021). Spatial and temporal analysis of hospitalized dengue patients in Bandung: demographics and risk. Trop Med Health.

[65] Garjito TA (2020). Stegomyia indices and Risk of Dengue Transmission: a lack of correlation. Front Public Heal.

[66] Vincenti-Gonzalez MF, et al. Spatial analysis of Dengue Seroprevalence and modeling of transmission risk factors in a Dengue Hyperendemic City of Venezuela. ” PLoS Negl Trop Dis. Jan. 2017;11(1):e0005317. 10.1371/journal.pntd.0005317. 10.1371/journal.pntd.0005317 PMC5289626 28114342

[67] Yuan K, Chen Y, Zhong M, Lin Y, Liu L. “Risk and predictive factors for severe dengue infection: A systematic review and metaanalysis,” *PLoS One*, vol. 17, no. 4 April, pp. 1–18, 2022, 10.1371/journal.pone.0267186. 10.1371/journal.pone.0267186 PMC9012395 35427400

[68] Morin CW, Comrie AC, Ernst K (2013). Climate and dengue transmission: evidence and implications. Environ Health Perspect.

[69] Ali K, Ma’Rufi I. The relationship between rainfall and dengue hemorrhagic Fever incidence during 2009–2013 (case study at Grati and Tutur Sub-district, Pasuruan, Indonesia). IOP Conf Ser Earth Environ Sci. 2018;200(1). 10.1088/1755-1315/200/1/012031.

[70] Drakou K, et al. The effect of weather variables on mosquito activity: a snapshot of the main point of entry of Cyprus. Int J Environ Res Public Health. 2020;17(4). 10.3390/ijerph17041403. 10.3390/ijerph17041403 PMC7068582 32098137

[71] Ahmed T, Hyder MZ, Liaqat I, Scholz M (2019). Climatic conditions: conventional and nanotechnology-based methods for the control of mosquito vectors causing human health issues. Int J Environ Res Public Health.

[72] Ma M, Huang M, Leng P (2016). Abundance and distribution of immature mosquitoes in urban rivers proximate to their larval habitats. Acta Trop.

[73] Semenzato P, Bortolini L. Urban Heat Island Mitigation and Urban Green Spaces: testing a model in the City of Padova (Italy). Land. 2023;12(2). 10.3390/land12020476.

[74] Wimberly MC (2020). Land cover affects microclimate and temperature suitability for arbovirus transmission in an urban landscape. PLoS Negl Trop Dis.

[75] Li Y, et al. Urbanization increases Aedes albopictus Larval habitats and accelerates Mosquito Development and Survivorship. PLoS Negl Trop Dis. 2014;8(11). 10.1371/journal.pntd.0003301. 10.1371/journal.pntd.0003301 PMC4230920 25393814

[76] Tana S (2013). Building and analyzing an innovative community-centered dengue-ecosystem management intervention in Yogyakarta, Indonesia. Pathog Glob Health.

[77] Krishnamoorthy K, Khan AB. “Entomological surveillance of dengue vectors in Tamil,” *J. Entomol. Zool. Stud*, vol. 2, no. l, pp. 158–164, 2014.

[78] Dejenie T, Yohannes M, Assmelash T (2011). Characterization of mosquito breeding sites in and in the vicinity of Tigray Microdams. Ethiop J Health Sci.

[79] Cummings DAT, et al. The impact of the demographic transition on dengue in Thailand: insights from a statistical analysis and mathematical modeling. PLoS Med. 2009;6(9). 10.1371/journal.pmed.1000139. 10.1371/journal.pmed.1000139 PMC2726436 19721696

[80] Martina BEE, Koraka P, Osterhaus ADME (2009). Dengue virus pathogenesis: an integrated view. Clin Microbiol Rev.

[81] Guha-Sapir D, Schimmer B (2005). Dengue Fever: new paradigms for a changing epidemiology. Emerg Themes Epidemiol.

[82] Liu-Helmersson J, Rocklöv J, Sewe M, Brännström Ã. “Climate change may enable Aedes aegypti infestation in major European cities by 2100,” *Environ. Res*, vol. 172, no. December 2018, pp. 693–699, 2019, 10.1016/j.envres.2019.02.026. 10.1016/j.envres.2019.02.026 30884421

[83] Salim MF, Syairaji M. “Time-Series Analysis of Climate Change Effect on Increasing of Dengue Hemorrhagic Fever (DHF) Case with Geographic Information System Approach in Yogyakarta, Indonesia,” in *International Proceedings the 2Ed International Scientific Meeting on Health Information Management*, 2020, vol. 5, pp. 248–256.

[84] Butterworth MK, Morin CW, Comrie AC (2017). An analysis of the potential impact of climate change on dengue transmission in the southeastern United States. Environ Health Perspect.

[85] Valdez LD, Sibona GJ, Diaz LA, Contigiani MS, Condat CA. “Effects of rainfall on Culex mosquito population dynamics,” *J. Theor. Biol*, vol. 421, no. March, pp. 28–38, 2017, 10.1016/j.jtbi.2017.03.024. 10.1016/j.jtbi.2017.03.024 28351704

[86] Clarke B, Otto F, Stuart-Smith R, Harrington L (2022). Extreme weather impacts of climate change: an attribution perspective. Environ Res Clim.

[87] Laporta GZ, Potter AM, Oliveira JFA, Bourke BP, Pecor DB, Linton YM. Global distribution of Aedes aegypti and Aedes albopictus in a Climate Change scenario of Regional Rivalry. Insects. 2023;14(1). 10.3390/insects14010049. 10.3390/insects14010049 PMC9860750 36661976

[88] Lamy K, Tran A, Portafaix T, Leroux MD, Baldet T (2023). Impact of regional climate change on the mosquito vector Aedes albopictus in a tropical island environment: La Réunion. Sci Total Environ.

[89] Harvey JA (2023). Scientists ’ warning on climate change and insects. Ecol Monogr.

[90] Silva NM, Santos NC, Martins IC. Dengue and zika viruses: epidemiological history, potential therapies, and promising vaccines. Trop Med Infect Dis. 2020;5(4). 10.3390/tropicalmed5040150. 10.3390/tropicalmed5040150 PMC7709709 32977703

[91] Kolimenakis A (2021). The role of urbanisation in the spread of aedes mosquitoes and the Diseases they transmit—a systematic review. PLoS Negl Trop Dis.

[92] Wilder-Smith A. “Dengue infections in travellers,” *Paediatr. Int. Child Health*, vol. 32, no. SUPP1, pp. 28–32, 2012, 10.1179/2046904712Z.00000000050. 10.1179/2046904712Z.00000000050 PMC3381444 22668447

[93] Tesema GA, Tessema ZT, Heritier S, Stirling RG, Earnest A. A systematic review of joint spatial and spatiotemporal models in Health Research. Int J Environ Res Public Health. 2023;20(7). 10.3390/ijerph20075295. 10.3390/ijerph20075295 PMC10094468 37047911

[94] Wongkoon S, Jaroensutasinee M, Jaroensutasinee K. “Distribution, seasonal variation & dengue transmission prediction in Sisaket, Thailand,” *Indian J. Med. Res*, vol. 138, no. SEP, pp. 347–353, 2013. PMC3818597 24135179

[95] Schmitt J, Bönig J, Borggräfe T, Beitinger G, Deuse J (2020). Predictive model-based quality inspection using machine learning and Edge Cloud Computing. Adv Eng Informatics.

[96] Yang CC (2022). Explainable Artificial Intelligence for Predictive modeling in Healthcare. J Healthc Informatics Res.

